# Early CYP3A5 Genotype-Based Adjustment of Tacrolimus Dosage Reduces Risk of De Novo Donor-Specific HLA Antibodies and Rejection among CYP3A5-Expressing Renal Transplant Patients

**DOI:** 10.3390/diagnostics14192202

**Published:** 2024-10-02

**Authors:** Kristina Schönfelder, Birte Möhlendick, Ute Eisenberger, Andreas Kribben, Winfried Siffert, Falko M. Heinemann, Anja Gäckler, Benjamin Wilde, Justa Friebus-Kardash

**Affiliations:** 1 Department of Nephrology, University Hospital Essen, University of Duisburg-Essen, 45147 Essen, Germany; kristina.schoenfelder@uk-essen.de (K.S.); ute.eisenberger@uk-essen.de (U.E.); andreas.kribben@uni-due.de (A.K.); anja.gaeckler@uk-essen.de (A.G.); benjamin.wilde@uk-essen.de (B.W.); 2 Institute of Pharmacogenetics, University Hospital Essen, University of Duisburg-Essen, 45147 Essen, Germany; birte.moehlendick@uk-essen.de (B.M.); winfried.siffert@uk-essen.de (W.S.); 3 Institute for Transfusion Medicine, Transplantation Diagnostics, University Hospital Essen, University of Duisburg-Essen, 45147 Essen, Germany; falko.heinemann@uk-essen.de

**Keywords:** *CYP3A5* polymorphism, tacrolimus dosage, tacrolimus trough levels, anti-HLA antibodies, DSAs, renal transplant rejection, renal transplant, calcineurin inhibitor nephrotoxicity

## Abstract

Background/Objectives: Our previous retrospective single-center cohort study found, at 3-year follow-up, a trend toward low tacrolimus trough levels and an increased risk of de novo donor-specific anti-HLA antibodies (DSAs) and of antibody-mediated rejection (ABMR) in CYP3A5-expressing patients. Determining CYP3A5-expression status immediately after renal transplant would allow early genotype-based dosage adjustment of tacrolimus and might prevent the occurrence of de novo DSAs and ABMR, improving transplant outcome. Methods: 160 renal allograft recipients who underwent renal transplant at the University Hospital Essen between May 2019 and May 2022 were genotyped for the *CYP3A5* rs776746 polymorphism within the first two weeks after transplant, and genotype-based dose adjustment of tacrolimus was performed for the follow-up of 2 years. Results: CYP3A5 expression was detected in 33 (21%) of the 160 patients. Tacrolimus trough levels were similar in CYP3A5 expressers and nonexpressers over the entire 2-year follow-up period. However, we observed a trend toward slightly higher tacrolimus trough levels in CYP3A5 expressers, who, as expected, required tacrolimus dosages twice as high as did nonexpressers during follow-up. Calcineurin inhibitor (CNI) nephrotoxicity-free survival rates were comparable between CYP3A5 expressers and nonexpressers (*p* = 0.49). Rejection-free survival rates (*p* = 0.89), de novo anti-HLA antibody-free survival rates (*p* = 0.57) and de novo DSA-free survival rates (*p* = 0.61) did not differ between the two groups. Conclusions: Early detection of CYP3A5-expression status and resultant genotype-based adjustment of tacrolimus dosage after renal transplant protected patients from transplant rejection and de novo DSA formation and was not associated with increased incidence of CNI toxicity among CYP3A5 expressers.

## 1. Introduction

Tacrolimus as a first-choice calcineurin inhibitor (CNI) is essential for maintaining immunosuppression among renal allograft recipients, with great beneficial effects on rejection rates and short-term allograft survival. However, tacrolimus exhibits a narrow therapeutic index between toxicity and efficacy, and its administration is characterized by pharmacokinetic inter- and intrapatient variability [1]. Therefore, therapeutic drug monitoring based on body weight and titration according to target blood concentrations is routinely used to adjust the dosage of tacrolimus [1,2]. In recent years, the use of pharmacogenetics as a tool for choosing the appropriate starting dose of immunosuppressive drugs has gained popularity. During recent decades, extensive investigation of the pharmacogenetics of tacrolimus for use in the field of transplant medicine has resulted in the discovery of several functional polymorphisms in the main tacrolimus-metabolizing enzymes CYP3A5 and CYP3A4 [2]. Hence, tacrolimus is considered one of the most promising candidates for pharmacogenetics-guided approaches, complementing conservative drug monitoring strategies with information about recipient genotypes for tacrolimus-metabolizing enzymes [2].

The CYP3A5 enzyme is abundantly expressed in the liver and small intestines and is a key enzyme for converting tacrolimus to its metabolites via oxidative and reductive pathways [1,3]. The single nucleotide polymorphism rs776746 is the most widely and best investigated functional polymorphism in the CYP3A5 enzyme and is predominantly responsible for the interpatient variability of tacrolimus [1]. The transition of A to G at position 6986 causes a cryptic splice site, forming a nonfunctional CYP3A5 protein [1,4]. Thus, individuals who are homozygous for this genetic abnormality and who harbor two aberrant alleles (CYP3A*3/*3) of the CYP3A5 gene are classified as CYP3A5 nonexpressers with impaired function of the CYP3A5 enzyme [1]. On the other hand, recipients with at least one wild-type allele (CYP3A*1/*3 or CYP3A*1/*31) are referred to as CYP3A5 expressers, with normal functioning of the CYP3A5 enzyme [1]. The prevalence of CYP3A5 expression among white populations is low but may reach 50% to 70% among Asian and Black patients [5]. CYP3A5 expressers who exhibit enhanced metabolic activity of the CYP3A5 enzyme are characterized as fast metabolizers, whereas CYP3A5 nonexpressers who express only a low amount of the functional CYP3A5 enzyme are considered slow metabolizers [1]. The effect of CYP3A5-expression status on the tacrolimus dose requirement has been consistently demonstrated by a great number of studies evaluating the transplant of kidneys and other solid organs into adult and pediatric patients of various ethnicities [2,6,7,8]. CYP3A5 expressers require 50% to 100% higher dosages of tacrolimus and attain target blood concentrations of the drug more slowly than do nonexpressers [2,9]. A meta-analysis involving European and Asian populations found significantly lower concentration-to-dosage ratios (C/D ratio) among CYP3A5 expressers than among nonexpressers at any posttransplant period [1]. Consequently, the guideline of the Clinical Pharmacogenetics Implementation Consortium (CPIC) recommends that the initial dosage of tacrolimus should be 1.5 to 2 times higher for CYP3A5 expressers than for nonexpressers [9].

The association between the CYP3A5 genetic variant and renal allograft rejection and outcome is controversial, as shown by several studies [8]. The latest meta-analysis could not confirm a correlation between CYP3A5-expression status and the development of acute rejection events or poorer renal allograft survival in European populations [10]. Indeed, in our previous retrospective study, the CYP3A5 variant was an independent risk factor for the development of de novo donor-specific antibodies (DSAs) and antibody-mediated rejection (ABMR) among renal allograft recipients [11]. On the basis of our findings, we hypothesized that tailoring tacrolimus dosage in accordance with CYP3A5 genotype might be a potentially helpful approach for optimizing tacrolimus dosing and exposure and for protecting renal allograft recipients from the appearance of de novo DSAs and from rejection attributed to underimmunosuppression [11].

Our study examined the effect of CYPA5 genotype-based dosage adjustment of tacrolimus directly after transplant on the course of tacrolimus trough levels and dosage requirements and on relevant clinical outcome parameters such as inclusive rejection events, CNI nephrotoxicity, and development of de novo DSAs in 160 renal allograft recipients who were followed up for 2 years after transplant.

## 2. Materials and Methods

### 2.1. Study Population

**Figure 1:**
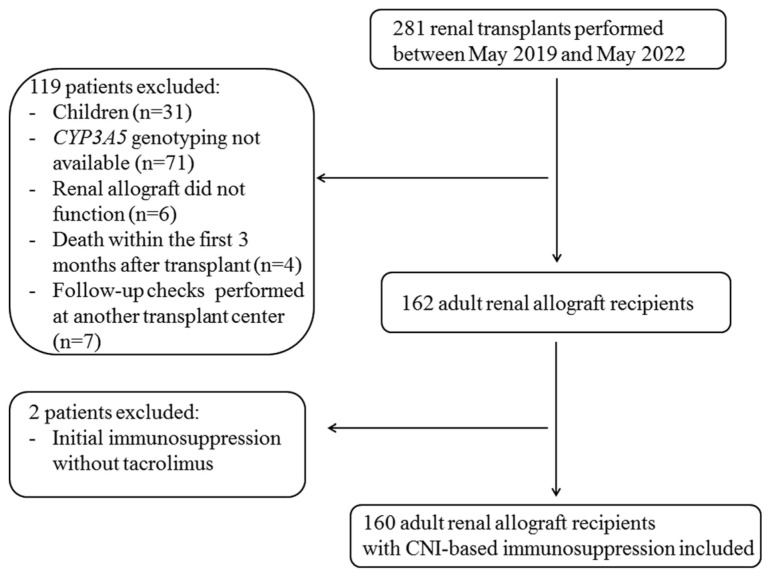
Study population flow chart with inclusion and exclusion criteria. CNI, calcineurin inhibitor.

Overall, 281 patients received renal transplants between May 2019 and May 2022 at our center (Figure 1), 31 of whom were excluded from this study because they were pediatric recipients. An additional 71 adult patients were excluded because the results of genetic testing for the *CYP3A5* genotype were not available (Figure 1). Furthermore, four patients who died within the first 3 months after transplant, six patients whose renal allografts failed to function, and seven patients who were followed up at another transplant center were excluded (Figure 1). Thus, 160 adult renal allograft recipients who underwent renal transplant and follow-up control visits at our transplant center and in whom *CYP3A5* genotyping was performed early after transplant were included (Figure 1). We set up a pilot study including all adult renal allograft recipients who were available in our transplant center from the introduction of routine *CYP3A5* genotyping in May 2019 and achieved a complete follow-up of two years and fulfilled the inclusion criteria. Due to the follow-up of at least two years post-renal transplant asked for renal allograft recipients in our pilot study, the inclusion period was restricted from May 2019 (start of the genotyping in clinical routine) to May 2022. The majority of patients were Caucasian (153 (96%) of 160 patients), one patient (0.6%) was of African origin, and 5 (3%) patients were Indian. Genotyping to determinate CYP3A5 status was performed within the first two weeks after renal transplant for 160 adult renal allograft recipients who received allografts between May 2019 and May 2022 at the University Hospital Essen and were treated with CNI-based immunosuppressive maintenance therapy. CYP3A5-expression status was determined within the two first weeks after transplant, was added to the diagnosis list, and was taken into account for subsequent genotype-based adjustment of CNI dosages. Renal allograft recipients were followed up for two years at the University Hospital Essen. Attending physicians at our transplant center were required to adhere to institution-specific target tacrolimus through levels of 7 to 9 ng/mL during the first 3 months and 5 to 7 ng/mL thereafter. The results of tacrolimus trough level measurements were provided within 4 h after taking blood of patients, and consequently, adaptation of tacrolimus dosage was performed on the same day of the clinical visit. In the case of CYP3A5 expressers, attending physicians were guided by target tacrolimus trough levels lying between 6–8 ng/mL after 3 months after transplant and subsequently prescribed a higher tacrolimus dosage to CYP3A5 expressers than to nonexpressers in order to achieve these target trough levels of tacrolimus. Tacrolimus trough levels under 5 ng/mL were not accepted for CYP3A5 expressers, and dosage adjustment was performed immediately. This strategy of *CYP3A5* genotype-based tacrolimus dosage adjustment was pursued for the entire period of follow-up of two years. Follow-up was terminated when patients were switched from CNIs to other immunosuppressive drugs. After follow-up, the following renal allograft outcome variables were analyzed retrospectively: rejection events, development of de novo anti-human leukocyte antigen (anti-HLA) antibodies and de novo anti-DSAs, occurrence of CNI nephrotoxicity, and estimated glomerular filtration rate (eGFR) course. All patients provided written informed consent before CYP3A5 genotyping. This study was approved by the institutional ethics board (19-9071-BO).

Clinical and laboratory data of allograft function were accessed by chart review of the electronic medical records for the first two years after transplant. Induction treatment with thymoglobulin was performed only for those patients who were considered to be at high immunological risk because their panel-reactive antibody (PRA) levels were higher than 25% or because they had previously undergone renal transplant. Recipients undergoing ABO-incompatible transplant were treated before transplant with a single 500 mg dose of intravenous (i.v.) rituximab, underwent immunoadsorption, and received i.v. immunoglobulin. Maintenance immunosuppression therapy consisted of CNIs, mycophenolate mofetil (MMF) or mycophenolic acid (MPA), and steroids according to the standard-of-care protocol at our transplant center. All patients except one received tacrolimus twice daily; the other was treated with an extended-release formulation of tacrolimus administered once daily.

Delayed graft function was defined in our study as a state of suboptimal function of the renal allograft within the first week after transplant requiring hemodialysis therapy [12]. All documented rejection episodes were biopsy-proven. Biopsies were performed for cause only during this study period, and samples were analyzed according to the latest available Banff grading criteria [13]. Experienced nephropathologists examined all renal transplant specimens with light microscopy and immunohistochemical analyses. The eGFR was calculated with the Chronic Kidney Disease Epidemiology Collaboration (CKD-EPI) equation [14].

In our transplant center, tacrolimus trough levels were routinely monitored weekly within the first 3 months after transplant and then at months 6, 9, and 12 and at least twice annually thereafter. Corresponding daily doses of tacrolimus were obtained from the medical records. Trough levels of tacrolimus were measured with chemiluminescent microparticle immunoassay (CMIA, Architect Tacrolimus, Abbott Diagnostics, Lake Forest, IL, USA). “The laboratory of the University Hospital Essen performing the chemiluminescent microparticle immunoassays (CMIA, ARCHITECT Tacrolimus 1L77©, Abbott, North Chicago, IL, USA) to quantify tacrolimus trough levels is an accredited laboratory. The test provides a measurable range between 2 and 30 ng/mL. In terms of quality management for chemiluminescent microparticle immunoassays, our laboratory takes part in interlaboratory tests in the context of the DIN EN ISO 15189 [15] program according to the recommendations of the “Bundesärztekammer”. Our laboratory also regularly performs internal quality controls”. The C/D ratio of tacrolimus, defined as the concentration-to-dosage ratio of tacrolimus, was calculated as the ratio of tacrolimus blood concentration to daily dosage. The intra-patient variability (IPV) was calculated in accordance with previous literature [16] for each renal allograft recipient as a quotient of the standard deviation of tacrolimus trough level measurements obtained over the follow-up of two years and the mean of tacrolimus trough level measurements obtained over the follow-up of two years. Then, the quotient was multiplied by 100.

### 2.2. HLA Typing of Recipients and Donors

For HLA typing of recipients and donors, we used either spin columns (Qiagen, Hilden, Germany) or an automated system based on magnetic separation technology (Chemagic, Chemagen PerkinElmer, Baesweiler, Germany) to isolate DNA from peripheral blood samples taken from patients and donors. HLA class I (HLA-A, -B, -C) and II (HLA-DRB1, -DQB1) typing was performed at the first-field resolution level with sequence-specific primers, the polymerase chain reaction sequence-specific amplification (PCR-SSP) method, or alternatively with sequence-specific oligonucleotides (LABType SSO method, both methods provided by One Lambda/Thermo Fisher Inc., Canoga Park, CA, USA) [17]. Second-field typing for selected high-resolution HLA alleles and serological equivalents was performed according to established Eurotransplant procedures [18]. HLA-DP typing and HLA-DQA typing were not performed, and HLA-DP– and HLA-DQA–specific antibodies were excluded from further analysis with respect to putative donor specificity of anti–HLA-DP and -DQA antibodies.

### 2.3. HLA Antibody Detection and Specification

All patients were screened for anti-HLA class I and II antibodies before transplant. Pretransplant patient sera collected closest to the date of transplant were used for screening. Pretransplant sensitization status was determined for all patients with the standard immunoglobulin G (IgG) complement-dependent cytotoxicity (CDC) test with or without the addition of dithiothreitol (DTT) to exclude antibodies of the IgM isotype. In addition, all patients were tested with a Luminex-based LABScreen Mixed bead assay (One Lambda, Thermo Fisher Scientific, Inc., Waltham, MA, USA) to identify antibodies against HLA classes I and II. In a broadly accepted step-by-step analysis [19], the serum anti-HLA antibodies with positive results on the LABScreen Mixed bead assay were subsequently tested specifically for HLA-A, -B, -C, -DR, -DP, and -DQ with LABScreen single-antigen bead (SAB) assays (One Lambda, Thermo Fisher Scientific Inc.) according to the manufacturer’s instructions. All beads with normalized median fluorescence intensity (MFI) values higher than 1000 were considered positive for anti-HLA antibodies. To address the potential effects of interfering antibodies or prozone effects on our MFI analyses, we generally analyzed patient samples after multiple freezing and thawing cycles (at least twice for each sample). We also retested HLA-DSA–positive sera after treatment with ethylenediaminetetraacetic acid (EDTA) by using SAB assays for HLA class I, II, or both in a single-batch confirmatory analysis [20]. Samples were measured with Luminex 100 or 200 machines and analyzed with HLA Fusion software version 4.4 (One Lambda/Thermo Fisher).

The results of pretransplant lymphocytotoxic T-cell crossmatches (CDC crossmatch) were negative for all recipients. Anti-HLA–antibody status after transplant was monitored at months 3, 6, and 12 after transplant and annually thereafter. Additional screening was performed in cases of allograft dysfunction. For the current study, de novo anti-HLA antibodies were detected at the earliest, 4 weeks after renal transplant with the same step-by-step Luminex HLA-antibody screening procedure that was used for pretransplant analysis. Again, all posttransplant sera were also examined with a complement-dependent lymphocytotoxicity test (CDC).

### 2.4. CYP3A5 Genotyping

DNA was isolated from samples of peripheral whole blood obtained from renal allograft recipients, and these samples were subjected to Polymerase chain reaction (PCR) for CYP3A5 rs776746 genotyping under the following conditions: 95 °C for 5 min; 38 cycles at 95 °C for 30 s, at 60 °C for 30 s, and at 72 °C for 30 s; and final elongation for 10 min at 72 °C (forward primer, 5′ TGTACCACCCAGCTTAACGA 3′; reverse primer, 3′ TTGTACGACACACAGCAACCT 5′). Genotyping by pyrosequencing was performed with a PyroMark Q96 MD instrument (Qiagen, Hilen, Germany) with the sequencing primer 5′ GCTCTTTTGTCTTTCA 3′ according to the manufacturer’s instructions. The Hardy-Weinberg equilibrium (HWE) was calculated with Pearson’s χ^2^ goodness-of-fit test, and genotypes were considered deviant from the HWE at a significance level of *p* < 0.05. CYP3A5 rs776746 results were within the HWE.

### 2.5. Statistical Analysis

Categorical variables were presented as numbers and percentages. The two-tailed χ^2^ test was used for comparisons between the two groups of recipients. Continuous variables were expressed as medians with ranges or interquartile ranges and were compared by using the Mann-Whitney test. We used one-way ANOVA to compare tacrolimus trough levels and tacrolimus dosages between CYP3A5 expressers and nonexpressers at various time points. Kaplan-Meier survival curves were created and compared with the log-rank test to evaluate differences between CYP3A5 expressers and nonexpressers. For all tests, statistical significance was set at the level of *p* < 0.05. All calculations were performed with GraphPad Prism version 6 (GraphPad Software, Inc., La Jolla, CA, USA) and IBM SPSS Statistics version 23 (IBM Corp., Armonk, NY, USA).

## 3. Results

### 3.1. Patient Characteristics

This study population consisted of 160 adult renal allograft recipients who underwent transplant between May 2019 and May 2022 at the University Hospital Essen, received CNI-based immunosuppressive therapy, and were screened for the CYP3A5 genotype immediately within two weeks after transplant (Figure 1).

The baseline demographic characteristics of this study cohort are presented in Table 1. The median age of the entire cohort was 52 years; 56 (35%) of the 160 patients were women (Table 1). As shown in Table 1, 26 (16%) recipients had previously undergone renal transplant, and 67 recipients exhibited preformed anti-HLA antibodies (42%) recipients. Most renal allografts (80%) were derived from deceased donors (Table 1). All renal allograft recipients were treated with antimetabolites (such as MMF or MPA) and steroids according to the standard-of-care protocol for the maintenance of immunosuppression, and 144 (90%) of the 160 recipients underwent induction therapy consisting of the interleukin-2 receptor antagonist basiliximab (Table 1).

Of the 160 renal allograft recipients, 33 (21%) were CYP3A5 expressers and 127 (79%) were nonexpressers (Table 1). Comparison of the two groups found no relevant differences in most baseline characteristics (Table 1). However, the proportion of patients with one or two HLA class I mismatches was significantly higher among the expressers than among the nonexpressers (Table 1).

### 3.2. Genotype-Based Adjustment of Calcineurin Inhibitor Dosage Led to Comparable Tacrolimus Trough Levels among CYP3A5 Expressers and Nonexpressers, Whereas the Tacrolimus Dosage Requirement of the Expressers Was Twice as High as That for the Nonexpressers over the 2-Year Follow-Up Period

**Figure 2:**
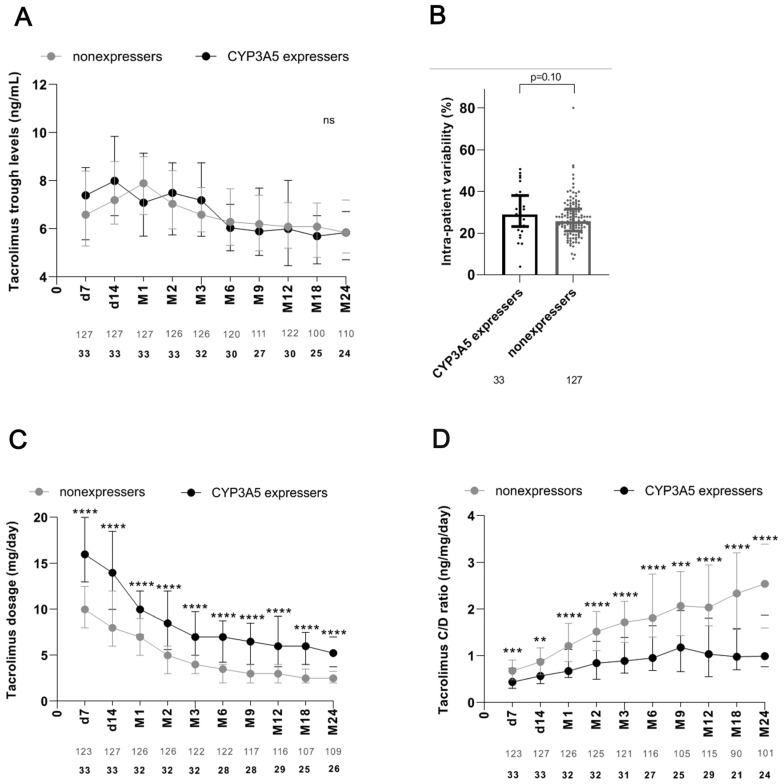
Quantification of tacrolimus trough levels and tacrolimus dosages after determination of CYP3A5 status immediately after renal transplant and subsequent genotype-based tacrolimus dosage adjustment for the follow-up period of 2 years. (**A**) Tacrolimus trough levels for CYP3A5 expressers and nonexpressers at indicated time points during 2-year follow-up after renal transplant. (**B**) Comparison of intra-patient variability of tacrolimus trough levels over the follow-up period of two years between 33 CYP3A5 expressers and 127 nonexpressers. (**C**) Tacrolimus dosages for CYP3A5 expressers and nonexpressers at indicated time points during 2-year follow-up after renal transplant. (**D**) Tacrolimus concentration-to-dose ratios (C/D ratios) for CYP3A5 expressers and nonexpressers at indicated time points during 2-year follow-up after renal transplant. Data are presented as medians with an interquartile range. **, *p* = 0.01; ***, *p* < 0.001; ****, *p* < 0.0001. C/D, ratio of concentration of tacrolimus to daily dosage of tacrolimus. d, day; M, month; ns, not significant.

Tacrolimus trough levels and daily tacrolimus doses were recorded at specific time points during the follow-up period of 2 years (Figure 2). With regard to tacrolimus trough levels, we found no statistically significant differences between the CYP3A5 expressers and nonexpressers (Figure 2A). At days 7 and 14 and at months two and three, we even saw a trend toward higher tacrolimus trough levels among the expressers than among the nonexpressers (Figure 2A). Comparison of intra-patient variability of tacrolimus trough levels over the follow-up period of two years between CYP3A5 expressers and nonexpressers indicated any significant differences (Figure 2B). Over the entire two-year follow-up period, CYP3A5 expressers required tacrolimus dosages twice as high as those required by nonexpressers (Figure 2C). Consequently, the corresponding tacrolimus C/D ratios were much lower among CYP3A5 expressers than among nonexpressers at all follow-up timepoints (Figure 2D). In general, from the sixth month after transplant forward, the tacrolimus dosage requirement for both groups was lower than that required during the first months of the postransplant period, and the tacrolimus trough levels of both groups stabilized at an average concentration of 6 ng/mL for both expressers and nonexpressers (Figure 2).

### 3.3. Comparable Renal Allograft Outcomes, in Particular Development of Rejection and De Novo DSAs, for CYP3A5 Expressers and Nonexpressers after an Early Genotype-Based Adjustment of Calcineurin Inhibitor Dosage

**Figure 3:**
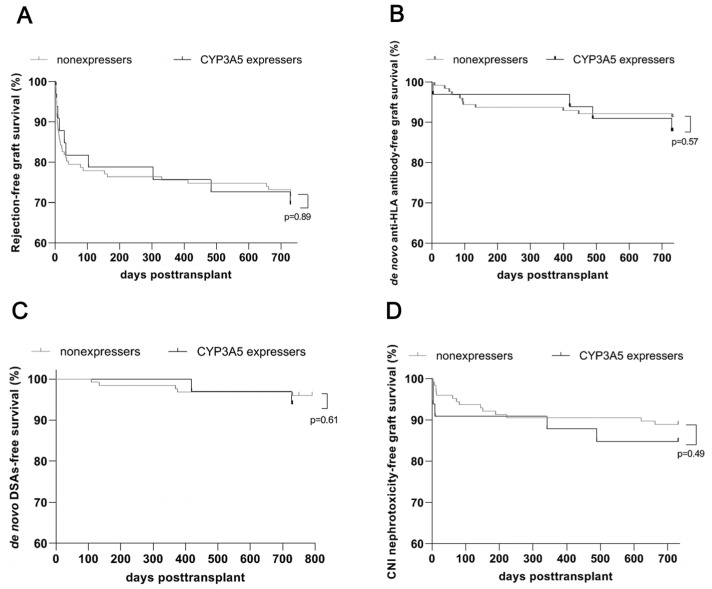
Renal allograft outcome among 33 CYP3A5 expressers and 127 nonexpressers at follow-up of 2 years after renal transplant with an early determination of CYP3A5 genotype and subsequent genotype-guided adjustment of tacrolimus-based immunosuppression. (**A**) Biopsy-proven rejection-free renal allograft survival rates (including Banff categories 2, 3, and 4) among renal transplant patients in relation to CYP3A5 genotype during 2-year follow-up after transplant. (**B**) Renal allograft survival rates for development of de novo anti-HLA antibodies according to CYP3A5 genotype during 2-year follow-up after transplant. (**C**) Renal allograft survival rates for development of de novo DSAs according to CYP3A5 genotype during 2-year follow-up after transplant. (**D**) Renal allograft survival rates for appearance of biopsy-proven calcineurin inhibitor–associated nephrotoxicity among renal transplant patients in relation to CYP3A5 genotype during 2-year follow-up after transplant. Anti-HLA, anti-human leukocyte antigen; CNI, calcineurin inhibitor; DSA, donor-specific antibody.

Table 2 illustrates the comparison of various renal allograft outcome variables between CYP3A5 expressers and nonexpressers 2 years after transplant. Delayed graft function occurred in 33 (21%) of our renal allograft recipients, but this outcome was not associated with the CYP3A5 genotype (Table 2). Biopsy-proven allograft rejection was documented for 46 (29%) patients (Table 2). The number of all rejection events occurring during the 2-year follow-up period was comparable between expressers and nonexpressers (Table 2), as were rejection-free survival rates (Figure 3A). During the relatively short follow-up period of 2 years after renal transplant, cellular and borderline rejections predominated in our study population (Table 2). The frequencies of Banff Diagnostic Category 3 and 4 rejections were similar between CYP3A5 expressers and nonexpressers (Table 2). Antibody-mediated rejections were rare, affecting two CYP3A5 expressers and one nonexpresser (Table 2).

In our cohort, de novo anti-HLA antibodies developed after renal transplant in 4 of 33 (12%) expressers and 12 of 127 (10%) nonexpressers. Similarly, we found no statistically significant differences between the two groups in de novo anti-HLA antibody-free survival (Figure 3B). We also found similar de novo DSA-free survival rates for expressers and nonexpressers (Figure 3C).

Biopsies of renal allografts found no significant differences in CNI nephrotoxicity between CYP3A5 expressers and nonexpressers (Table 2). Analysis of CNI-free survival according to CYP3A5 expresser status confirmed these results (Figure 3D).

The eGFR values detected at the end of the 2-year follow-up did not differ significantly between CYP3A5 expressers and nonexpressers (Table 2). Additionally, the percentage of allograft recipients experiencing a decrease in eGFR below 30 mL/min/1.73 m^2^ was similar between the two groups, a finding suggesting no relationship between renal transplant function and CYP3A5 genotype (Table 2).

## 4. Discussion

Our single-center study involving 160 renal allograft recipients whose tacrolimus dosage was adjusted according to CYP3A5 genotype found similar tacrolimus trough levels among CYP3A5 expressers and nonexpressers within the two-year follow-up period after transplant. However, CYP3A5 expressers required tacrolimus dosages 2-fold higher during follow-up and exhibited significantly lower C/D ratios than did nonexpressers. We detected no significant differences between expressers and nonexpressers in the incidence of delayed graft function, rejection, CNI nephrotoxicity, and development of de novo anti-HLA antibodies and de novo DSAs after the follow-up period of 2 years. Renal allograft function was also well preserved in both groups during follow-up.

Pasari et al. performed a retrospective analysis with a study design similar to that used in our study [21]. Their study, which involved 25 renal allograft recipients given CYP3A5-guided immunosuppressive therapy, analyzed tacrolimus trough concentrations and clinical renal allograft outcomes at 6 to 12 months after renal transplant. Of the recipients, 40% carried the CYP3A5-expresser variant [21]. In agreement with our findings, they observed higher tacrolimus C/D ratios among nonexpressers than among expressers. They also found no disturbance in renal allograft function and no increased frequency of rejection among expressers after genotype-guided adjustment of tacrolimus dosage [21]. In contrast to our study, they performed CYP3A5 genotype testing before renal transplant, whereas we performed genotyping within the first two weeks after transplant [21]. In addition, their study involved a small number of transplant recipients and limited follow-up, to a maximum of 12 months. Thus, it is difficult to draw concrete conclusions from their study [21].

Recently published prospective randomized multicenter clinical trials investigating the impact of genotype-based tacrolimus dosing used study designs distinct from ours [22,23,24,25]. All of those studies randomly assigned renal allograft recipients to treatment either according to the CYP3A5 genotype determined before transplant or according to a standard daily regimen [22,23,24,25,26,27,28,29]. CYP3A5 expressers receiving genotype-guided treatment were provided with higher tacrolimus dosages than did nonexpressers [22,23,24,25]. Then, all patients in the CYP3A5-guided dosing group were compared with corresponding patients from the standard dosing group in terms of tacrolimus trough levels and allograft outcomes without differentiation between expressers and nonexpressers [22,23,24,25]. The French randomized trial involving 280 transplant patients described that a larger proportion of patients in the genotype-guided dosing group whose tacrolimus concentrations were at a steady state within the therapeutic range within the first 3 days after transplant tended to exhibit therapeutic tacrolimus concentrations earlier and without dose adjustments than did the conventional dosing group [22]. An additional prospective randomized study from the Netherlands involving 240 recipients of renal transplants from living donors could not replicate the results of the French trial [23]. Despite substantial differences in study design between our study and these two trials, we saw that CYP3A5 genotype-guided tacrolimus dosage adjustment enabled the attainment of similar target concentrations for all renal allograft recipients, regardless of CYP3A5 status, a finding that parallels the results of the two above-mentioned randomized trials [22,23,24,25,26,27,28,29]. However, our previous retrospective study involving renal allograft recipients who received standard weight-based tacrolimus dosage adjustments and might be considered a reference cohort found a tendency toward lower tacrolimus trough levels among CYP3A5 expressers than among nonexpressers, a finding suggesting insufficient immunosuppression among CYP3A5 expressers [11]. Similar findings were obtained from previous studies reporting that target tacrolimus concentrations were achieved after a significant delay among CYP3A5 expressers undergoing conventional drug monitoring; therefore, these recipients may have been exposed to subtherapeutic tacrolimus levels for a longer time period than were nonexpressers [30,31].

Nevertheless, the findings of the prospective randomized clinical trials and the subsequent systemic reviews agreed that genotype-based tacrolimus dosing directly after renal transplant was not superior to the classic weight-based tacrolimus dosing strategy for clinical endpoints such as delayed graft function, acute rejection, CNI nephrotoxicity, and allograft loss [22,23,24,25,26,27,28,29]. Diverse reasons might underlie this absence of benefit from genotype-based tacrolimus dose adjustment. Because of the relatively small number of allograft recipients and the short follow-up periods (a maximum of 5 years), the statistical power of the randomized prospective trials was too low to detect significant differences in clinical outcomes [22,23,24,25,26,27,28,29]. The frequency of CYP3A5 expresser status differs according to the ethnic origin of study cohorts [1,2]. The participants in the prospective trials were white and exhibited a lower prevalence of the CYP3A5-expresser variant than did Asian participants [1,2,22,23,24,25,26,27,28,29]. The evidence of an elevated risk of rejection among CYP3A5 expressers in Asian cohorts and a lack of rejection risk among CYP3A5 expressers in European cohorts supports the presumption that the low prevalence of CYP3A5 expression among Europeans may have contributed to an underestimation of the effects of CYP3A5-expression status and the positive effects of genotype-guided therapy on overall renal outcomes [8,10,22,23,24]. Otherwise, CYP3A5 genotype-based dosing may be more relevant for Asian than for European recipients.

Our study found no significant differences in estimated renal allograft outcomes between CYP3A5 expressers and nonexpressers who underwent therapeutic drug monitoring based on pharmacogenetics. In contrast to the recent prospective randomized clinical trials, our study did not include a control group receiving conventional drug monitoring [22,23,24,25,26,27,28,29]. Like our study, however, the corresponding clinical trials involved low-risk populations generally exhibiting a low frequency of rejection events, de novo DSAs, and allograft failures that might have led us to overlook potential differences [22,23,24,25,26,27,28,29]. Furthermore, potent immunosuppressive induction therapy and maintenance therapy regimens were given to our recipients and to recipients in the previously reported clinical trials; such regimens strongly reduce the risk of rejection and delayed graft function and mitigate the effects of tacrolimus underdosing in the early posttransplant period [22,23,24,25,26,27,28,29]. Thus, under these conditions, this study might have been unlikely to capture differences in renal allograft outcomes. The early and successful achievement of target tacrolimus concentrations with a genotype-based dosing approach might fade into the background and appear secondary and less important considering the current use of potent immunosuppressive agents. The administration of corticosteroids was found to induce CYP3A5 enzyme activity, in particular among nonexpressers, which would have accelerated tacrolimus metabolism and may have overshadowed the effects of CYP3A5-expression status in our study [32]. Moreover, several other meaningful environmental factors besides the CYP3A5 genotype affect target tacrolimus concentrations, such as gastrointestinal motility, adherence, pharmaceutical formulation (immediate release versus extended release), drug-drug interactions, and nutritional habits (food-drug interactions), as well as functional polymorphisms in other enzymes involved in tacrolimus metabolism [33,34].

In fact, comparison of CYP3A5 expressers with nonexpressers with respect to occurrence of acute antibody-mediated rejections (ABMR) and transplant failure revealed a statistically significant difference between the two groups. However, ABMR and transplant failure were very rare events that appeared after the follow-up of 2 years post-transplant in our renal allograft recipient cohort. Such disease processes, such as ABMR and, in particular, transplant failure, need many years to evolve and therefore mainly occur late after transplant. We think that the follow-up of two years was too short for the reliable detection of the development of ABMR events and transplant loss that might be attributable to the *CYP3A5* genotype-based tacrolimus dose adjustment. It is more likely that other acute events rather than *CYP3A5* genotype and *CYP3A5* genotype-based tacrolimus dose adjustment might be responsible for ABMR or transplant failure in a few recipients at early time points after transplant in our cohort. Thus, these statistically significant results are not fully supported by clinical relevance and should be interpreted with caution.

Therefore, rather than basing initial tacrolimus dosages on CYP3A5 genetic profiling alone, the incorporation of demographic and clinical factors and multiple genetic variants in a dosing algorithm may help to more accurately predict tacrolimus dose requirements [2]. In recent years, a few pharmacokinetic and polygenetic models have been validated [2]. Two recent studies showed that a polygenetic approach combining CYP3A5 and CYP3A4 genotyping and classifying renal allograft recipients as poor, intermediate, or fast metabolizers was beneficial in terms of predicting initial dosages of tacrolimus [35,36]. This research group of Lloberas et al. created a Population Pharmacokinetic Model using Bayesian prediction and integrating information on age, CYP3A5 and CYP3A4 genotype, and hematocrit levels for personalized tacrolimus dosing [37]. They evaluated the clinical applicability of their model in a prospective controlled randomized trial involving 90 renal allograft recipients separated into two study arms [37]. The control arm underwent tacrolimus dosage adjustment based on empirical calculations of body weight according to the manufacturer’s instructions, whereas the model arm underwent initial and maintenance tacrolimus dosage adjustments according to the Population Pharmacokinetic Model described above [37]. A significantly higher proportion of recipients in the model arm (using the Population Pharmacokinetic Model) exhibited target tacrolimus concentrations than did recipients in the control arm [37]. Steady-state tacrolimus concentrations were achieved significantly sooner and with fewer dosage adjustments within 90 days after transplant in the model arm than in the control arm [37]. This study offered no evidence of improvement in clinical outcomes when the initial tacrolimus dosage was determined according to the Population Pharmacokinetic Model [37]. As a result of these findings, the second consensus report on therapeutic drug monitoring of tacrolimus-personalized therapy strongly recommended integration of genetic information about genotypes CYP3A5*3 and CYP3A4*22 in future Population Pharmacokinetic Models [2].

Pharmacogenetic testing is a feasible and inexpensive approach that could be established as part of a standard care setting in transplant units. To date, the cost-effectiveness of preemptive pharmacogenetic testing of renal allograft recipients has not been well studied. The cost-effectiveness analysis conducted by Deininger et al. compared economic outcomes between CYP3A5 genotype-based dosing and standard care dosing among recipients of solid organ transplant with a follow-up of six months. Using a probabilistic sensitivity analysis, these researchers proposed the likelihood of a cost savings of 19.8% for CYP3A5 genotype-guided dosing of renal allograft recipients [38]. Future cost-effectiveness studies of long-term renal allograft outcomes comparing conventional standard care tacrolimus dosing with genotype-based dosing strategies and models are needed to lay the foundation for large-scale implementation of pharmacogenetic testing in the renal transplant setting.

With a special focus on CNI nephrotoxicity, we found that reliable tacrolimus concentrations in the therapeutic range tended to be more likely after CYP3A5 genotype-based dosage adjustment among CYP3A5 expressers than among nonexpressers, but CYP3A5 expressers were not at higher risk of CNI nephrotoxicity, as determined by allograft biopsy. In line with our data, the meta-analysis of Roja et al. could not confirm the relationship between CYP3A5-expression status and acute nephrotoxicity [8]. The results of the five published studies were conflicting in terms of the effect of the CYP3A5-expresser variant on chronic biopsy-proven CNI nephrotoxicity [8]. The largest study, performed by Kuypers et al. and involving 304 renal allograft recipients, observed an association between CYP3A5-expression status and a higher incidence of CNI nephrotoxicity [39]. The authors suggested that nephrotoxicity may have been provoked by a higher concentration of tacrolimus metabolites in transplanted kidneys because of accelerated intrarenal tacrolimus metabolism by activated functional CYP3A5 enzyme in expressers [39]. Intrarenal tacrolimus concentrations that did not properly match blood tacrolimus concentrations play a pivotal role in the development of CNI nephrotoxicity [6,40]. Therefore, it is conceivable that the occurrence of CNI nephrotoxicity may not depend on the CYP3A5 genotype of the recipient but rather on that of the donor [6]. The donor’s nonexpression genotype, reflecting low tacrolimus metabolism locally in the renal allograft, may result in the accumulation of tacrolimus and the mediation of nephrotoxicity, despite normal blood levels of tacrolimus. Additional research taking into account the CYP3A5 genotypes of the donors and the recipients is necessary for addressing this assumption regarding CNI nephrotoxicity.

Our study contains several limitations incorporating a retrospective single-center study design, a limited number of patients in particular among CYP3A5 expressers, a relative short follow-up period, especially for appropriate detection of ABMR and transplant failure events, the occurrence of missing data due to failure to attend clinical visits by some patients, and the lack of a suitable control group that did not undergo *CYP3A5* genotype-based tacrolimus dose adjustment.

Our single-center study highlighted that the CYP3A5 genotype-guided adjustment of Tacrolimus dosage for initial and maintenance immunosuppressive therapy is an efficient and safe option for achieving target tacrolimus levels among CYP3A5 expressers similar to those among nonexpressers and for preventing a higher occurrence of rejection and of de novo anti-HLA antibodies in this sensitive subgroup of renal allograft recipients. Our results support the opinion that further efforts should be made to complement standardized therapeutic drug monitoring of tacrolimus concentrations by the addition of pharmacogenetics and clinical and demographic factors for establishing tacrolimus dosing algorithms with greater predictive value. These changes may allow individual refinement of posttransplant immunosuppressive regimens, avoiding over- and underexposure to tacrolimus and moving toward personalized immunosuppressive treatment.

## Figures and Tables

**Table 1:** Baseline characteristics of 160 adults who underwent renal allograft transplant between May 2019 and May 2022 at the University Hospital Essen, were treated with calcineurin inhibitor-based immunosuppression, and underwent genotyping for CYP3A5 status.

	All Patients (*n* = 160)	CYP3A5 Expressers (*n* = 33)	Nonexpressers (*n* = 127)	RR (95%CI)	*p* Value
**Recipient**					
Age, median (range)	52 (19–76)	49 (23–76)	52 (19–77)		0.79
Sex, women, *n* (%)	56 (35)	9 (27)	47 (37)	0.74 (0.4–1.3)	0.3
Sex, men, *n* (%)	104 (65)	24 (73)	80 (63)	1.16 (0.9–1.4)	0.3
Previous transplants, *n* (%)	26 (16)	8 (24)	18 (14)	1.71 (0.8–3.4)	0.16
PRA, *n* (%)	8 (5)	1 (3)	7 (6)	0.55 (0.1–3.2)	0.56
Preformed anti-HLA antibodies, *n* (%)	67 (42)	12 (36)	55 (43)	0.84 (0.5–1.3)	0.47
Class I, *n* (%)	45 (28)	9 (27)	36 (28)	0.96 (0.5–1.7)	0.9
Class II, *n* (%)	43 (27)	6 (18)	37 (29)	0.62 (0.3–1.3)	0.21
Class I and II, *n* (%)	21 (13)	3 (9)	18 (14)	0.64 (0.2–1.8)	0.44
Cold ischemia time in min, median (range)	593 (85–10,404)	566 (103–1094)	598 (85–1404)		0.74
Warm ischemia time in min, median (range)	25 (11–48)	26 (11–46)	25 (11–48)		0.81
**Donor**					
Deceased donors, *n* (%)	128 (80)	26 (79)	102 (80)	0.98 (0.8–1.2)	0.85
Age, median (range)	56 (16–82)	58 (30–72)	55 (16–82)		0.29
Sex, women, *n* (%)	71 (44)	18 (55)	53 (42)	1.31 (0.9–1.8)	0.19
Sex, men, *n* (%)	89 (56)	15 (45)	74 (58)	0.78 (0.5–1.1)	0.19
ABO-incompatible transplant, *n* (%)	19 (12)	4 (12)	15 (12)	1. 03 (0.4–2.7)	0.96
**Immunosuppression at transplant**					
Interleukin-2 receptor antagonist, *n* (%)	144 (90)	29 (88)	115 (91)	0.97 (0.8–1.1)	0.65
ATG, *n* (%)	16 (10)	3 (9)	13 (10)	0.89 (0.3–2.6)	0.85
Tacrolimus extended-release formulation, *n* (%)	1 (1)	0 (0)	1 (1)	0 (0–11.4)	0.61
MMF/MPA, *n* (%)	160 (100)	33 (100)	127 (100)		
Steroids, *n* (%)	160 (100)	33 (100)	127 (100)		
**HLA mismatches**					
MM A/B, *n* (%)	133 (83)	31 (94)	102 (80)	1.17 (1.0–1.3)	0.06
HLA class I MM A/B: 1–2, *n* (%)	82 (51)	22 (67)	60 (47)	1.41 (1.0–1.9)	**0.05**
HLA class I MM A/B: 3–4, *n* (%)	52 (33)	10 (30)	42 (33)	0.92 (0.5–1.6)	0.76
MM DR, *n* (%)	108 (68)	26 (79)	82 (65)	1.22 (0.9–1.5)	0.12
HLA class II MM DR: 1, *n* (%)	71 (44)	16 (48)	55 (43)	1.12 (0.7–1.6)	0.59
HLA class II MM DR: 2, *n* (%)	37 (23)	10 (30)	27 (21)	1.43 (0.8–2.5)	0.27
**Causes of renal failure**					
1. Diabetic glomerulosclerosis, *n* (%)	16 (10)	5 (15)	11 (9)	1.75 (0.7–4.4)	0.27
2. Chronic glomerulonephritis, *n* (%)	8 (5)	2 (6)	6 (5)	1.28 (0.3–5.2)	0.75
3. Nephrosclerosis, *n* (%)	26 (16)	5 (15)	21 (17)	0.92 (0.4–2.1)	0.85
4. Polycystic kidney disease, *n* (%)	28 (18)	4 (12)	24 (19)	0.64 (0.2–1.6)	0.36
5. Tubulointerstitial nephritis, *n* (%)	32 (20)	7 (21)	25 (20)	1.08 (0.5–2.2)	0.85
6. Congenital anomalies, *n* (%)	16 (10)	3 (9)	13 (10)	0.89 (0.3–2.6)	0.85
7. Autoimmune diseases, *n* (%)	10 (6)	2 (6)	8 (6)	0.96 (0.2–3.7)	0.96
8. Reflux nephropathy/recurrent pyelonephritis, *n* (%)	8 (5)	0 (0)	8 (6)	0 (0–1.7)	0.14
9. TMA, *n* (%)	3 (2)	1 (3)	2 (2)	1.92 (0.3.11.1)	0.58
10. Other, *n* (%)	15 (9)	4 (12)	11 (9)	1.4 (0.5–3.8)	0.54

Anti-HLA, anti-human leukocyte antigen; ATG, anti-thymocyte globulin; CI, confidence interval; MM, mismatch; MMF, mycophenolate mofetil; MPA, mycophenolic acid; PRA, panel-reactive antibodies; RR, relative risk; TMA, thrombotic microangiography.

**Table 2:** Renal allograft outcome variables among 33 CYP3A5 expressers and 127 nonexpressers at follow-up of 2 years after renal transplant.

	All Patients (*n* = 160)	CYP3A5 Expressers (*n* = 33)	Nonexpressers (*n* = 127)	RR (95%CI)	*p* Value
Delayed graft function, *n* (%)	33 (21)	6 (18)	27 (21)	0.86 (0.4–1.8)	0.7
Biopsy, *n* (%)	73 (46)	19 (58)	54 (43)	1.35 (0.9–1.9)	0.12
>1 biopsy, *n* (%)	33 (21)	9 (27)	24 (19)	1.44 (0.7–2.7)	0.29
Rejection, Banff categories 2, 3, and 4, *n* (%)	46 (29)	10 (30)	36 (28)	1.07 (0.6–1.8)	0.82
Rejection Banff categories 2 and 4, *n* (%)	29 (18)	8 (24)	21 (17))	1.47 (0.7–2.9)	0.31
ABMR Banff category 2, *n* (%)	3 (2)	2 (6)	1 (1)	7.7 (1.0–57.4)	0.05
TCMR Banff categories 3 and 4, *n* (%)	44 (28)	9 (27)	35 (28)	0.99 (0.5–1.8)	0.97
TCMR Banff category 4, *n* (%)	21 (13)	4 (12)	17 (13)	0.91 (0.3–2.3)	0.85
Transplant failure, *n* (%)	5 (3)	3 (9)	2 (2)	5.77 (1.2–27.8)	0.03
eGFR CKD-EPI mL/min/1.73 m^2^ at 2 years after Tx, median (range)	54 (12–119)	58 (12–99)	54 (112–119)		0.36
eGFR < 30 mL/min/1.73 m^2^ at 2 years after Tx, median (range)	21 (13)	4 (12)	17 (13)	0.91 (0.3–2.3)	0.85
Death, *n* (%)	1 (1)	0 (0)	1 (1)	0 (0.14.4)	0.61
De novo anti-HLA antibodies, *n* (%)	17 (11)	4 (12)	13 (10)	1.18 (0.4–3.1)	0.75
Class I, *n* (%)	8 (5)	2 (6)	6 (5)	1.28 (0.3–5.2)	0.75
Class II, *n* (%)	11 (7)	3 (9)	8 (6)	1.44 (0.4–4.6)	0.57
De novo anti-HLA DSAs, *n* (%)	7 (4)	2 (6)	5 (4)	1.54 (0.4–6.5)	0.6
Class I, *n* (%)	3 (2)	1 (3)	2 (2)	1.92 (0.3–14.1)	0.58
Class II, *n* (%)	6 (4)	2 (6)	4 (3)	1.92 (0.4–8.5)	0.43
CNI nephrotoxicity, *n* (%)	19 (12)	5 (15)	14 (11)	1.37 (0.5–3.3)	0.51
Follow-up time in years, median (range)	2 (0.3–2)	2 (0.3–2)	2 (0.5–2)		0.22

ABMR, antibody-mediated rejection; anti-HLA, anti-human leukocyte antigen; CI, confidence interval; CKD-EPI, Chronic Kidney Disease Epidemiology Collaboration; CNI, calcineurin inhibitor; DSAs, donor-specific antibodies; eGFR, estimated glomerular filtration rate; RR, relative risk; TCMR, T cell–mediated rejection; Tx, transplant.

## Data Availability

The original contributions presented in the study are included in the article, further inquiries can be directed to the corresponding author.
